# A complex morphofunctional approach for zinc toxicity evaluation in rats

**DOI:** 10.1016/j.heliyon.2020.e03768

**Published:** 2020-04-21

**Authors:** Gennadii Piavchenko, Alexander Alekseev, Olga Stelmashchuk, Evgeniya Seryogina, Evgeny Zherebtsov, Elena Kuznetsova, Andrey Dunaev, Yuri Volkov, Sergey Kuznetsov

**Affiliations:** a I.M. Sechenov First Moscow State Medical University (Sechenov University), Russian Federation; b Pharmaceutical R&D Enterprise “Retinoids”, Russian Federation; c Orel State University named after I.S. Turgenev, Russian Federation; d University of Oulu, Finland; e School of Medicine and Trinity Translational Medicine Institute, The University of Dublin, Trinity College, Ireland

**Keywords:** Zinc succinate, Tissue and organ toxicity, Morphofunctional approach, Behavioral activity, Endogenous fluorescence, Experimental rats, Health sciences, Neuroscience, Psychiatry, Toxicology

## Abstract

Anthropogenic activity causes the introduction of zinc compounds into the biological cycle in mining and processing sites and its accumulation in organs and tissues, causing systemic toxicity. A cumulative effect of zinc is predominantly neurotoxic and it also affects the respiratory, cardiovascular and digestive systems. This study evaluates the effects of single-dose intragastric administration of 100 mg/kg zinc succinate on the structure and function of organs and tissues in male Wistar rats 1 month after treatment. The presented morphofunctional approach for the toxicity evaluation included the study of behavioral responses using the automated Laboras® complex, fluorescent spectral analysis of the NADH and FAD activity and histological evaluation of animal organs and tissues. The results of the behavioral activity assessment showed a significant decrease in animals’ motor activity, whereas the fluorescence spectra analysis demonstrated a decrease in coenzyme NADH without the reduction of FAD levels. We detected toxic and dystrophic changes in the cerebral cortex, heart, lungs and liver tissues. Our original multiparametric approach enables a comprehensive assessment of the long-term toxic effects of the metal salts such as zinc succinate, especially in the cerebral cortex at the doses much lower than the acute LD_50_ reported for the common zinc salts.

## 1 Introduction

It is well established that the chronic exposure of mammalian organisms to zinc salts induces toxic effects in nearly all organs and tissues, and especially in the central nervous system [1, 2]. According to the literature, the increase of the zinc and succinic acid concentration may lead to the development of neurodegenerative diseases of the central nervous system [3, 4]. It has been shown that the increase of zinc concentration in the body might cause dysfunctions of the immune system and provoke a deficiency in the content of other chemical compounds such as iron and copper. Over the extended time, excess of zinc substances may lead to dystrophic processes in the liver, pancreas and other digestive organs [5]. Industrial processes such as mining, processing and production of metal salts (e.g. zinc), contribute to their intensive release into the environment. They enter the atmosphere as a component of the technogenic dust, subsequently settling in the open water reservoirs as well as on the surface of soil and plants. In production areas, zinc content in biological objects exceeds acceptable levels and its further supply to the human organism through food chains leads, in turn, to accumulation in organs and tissues. There is evidence that chronic intoxication with zinc succinate in small doses causes an inhibition of the motor activity of animals, which is in line with the concept of the neurotoxic effect of such substances [6, 7].

**Figure 1:**
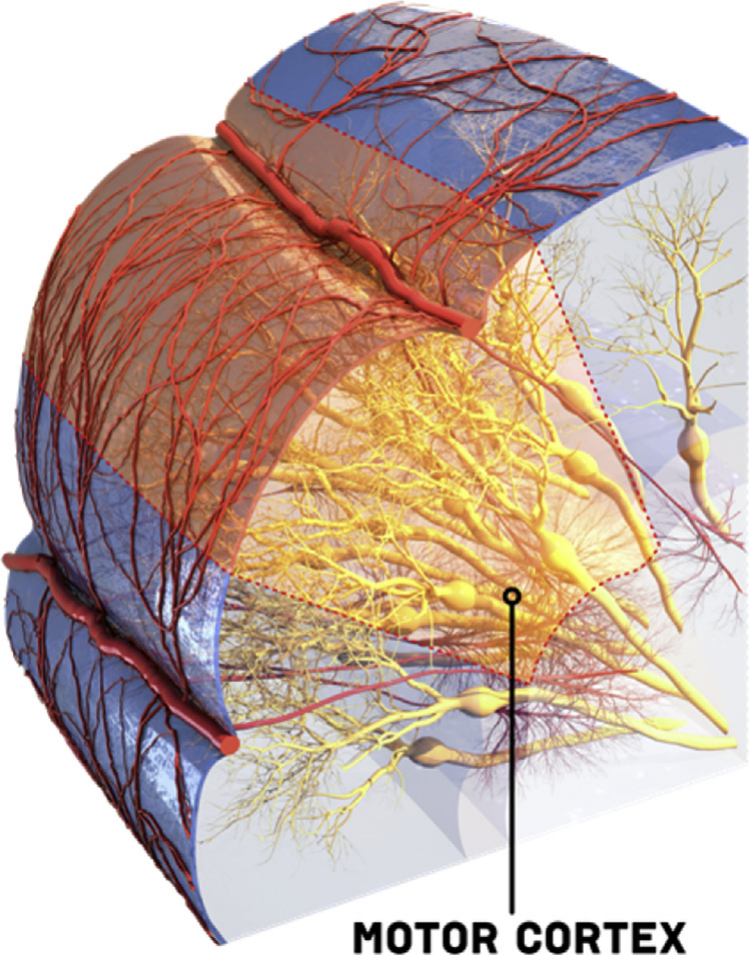
Rat brain motor cortex model.

Motor cortex in rats (Figure 1) is the main source of activating impulses to the motor neurons of the spinal cord, being the region of the motor response initiation. The signals sent by certain parts of the motor cortex stimulate the motor activity in one or another part of the body, indicating the presence of the activating effect projection zones. Structurally it represents an agranular type of the cortex and has the predominantly expressed III and V layers, which contain large and giant pyramid neurons. These cells send the impulses downstream for the signal transduction.

Morphological analysis of organs and tissues is the golden standard for the investigation of systemic toxicity. Along with the routine morphology, an effective method for evaluation of the chemical substance action on the organism is the evaluation of the behavioral reactions of animals with automated non-invasive systems. Another new approach is to analyze parameters of endogenous fluorescence of cells for metabolic parameters of brain structures *in vivo* by fluorescence spectroscopy [8, 9]. This method is based on the excitation of endogenous and exogenous biological tissue fluorophores and the subsequent registration of their fluorescence. Fluorescence spectroscopy technique is highly sensitive [10] and enables assessing both the intensity of metabolic processes and the state of oxygen metabolism in tissues.

In this study implementing a complex morphofunctional approach, we evaluated the behavioral and structural changes in the organs and tissues of male Wistar rats 1 month after intragastric administration of zinc succinate at a single dose of 100 mg/kg (an equivalent dose for human is 0.5 g/kg). Along with a significant decrease in motor activity, histological analysis revealed the presence of toxic and dystrophic processes in the cerebral cortex, heart, lungs and liver of the exposed animals. In parallel, fluorescence spectra tissue analysis demonstrated a decrease in its intensity for the coenzyme NADH without the reduction of FAD coenzyme levels. We believe that our original multiparametric approach offers a comprehensive assessment of the long-term toxic effects of the metal salts such as zinc succinate on organs and tissues including the cerebral cortex and that it is potentially applicable for studying a broader spectrum of pathological conditions hallmarked by neurodegeneration.

## 2 Materials and methods

### 2.1 Animals

The studies were performed on male 2-month-old Wistar rats. Animals were obtained from the animal breeding facility "Andreevka", a branch of the FGBUN "NCBMT FMBA" of the Russian Federation.

### 2.2 Regulatory and ethical standards

All procedures for the care of animals were performed in accordance with the SOPs of the Center for Preclinical Research, J.-s.c. “Retinoids”. The study was conducted in compliance with the standard "Principles of Good Laboratory Practice" [11] and in accordance with ARRIVE guidelines and the National Institutes of Health guide for the care and use of Laboratory animals (NIH Publications No. 8023, revised 1978). Manipulations with animals, as well as the maintenance conditions, were reviewed and approved by the Commission for Control over the Maintenance and Use of Laboratory Animals of Pharmaceutical R&D Enterprise “Retinoids”.

### 2.3 Conditions of animal maintenance and care

Rats were kept in open cages in separate rooms of the vivarium premises designed for small laboratory animals in controlled environmental conditions (20–26 °C and a relative humidity of 30–70%). The temperature and humidity in the rooms were monitored daily using automatic electronic thermometers and psychrometers. A 12-hour lighting cycle and a 10-times exchange of the room air volume per hour was maintained in the rooms.

Feeding of the animals was carried out using a standard balanced granulated chow for rodents (LLC “Laboratorkorm”, Russia) in accordance with the daily physiological needs [12]. Distilled deionized water was supplied *ad libitum*.

Upon arrival to the vivarium, the qualified veterinarian performed the overall health check of the animals and subsequently carried out daily inspection during a two-week quarantine period. Before the transfer to a clean zone and allocation to the experimental groups, the animals received a further clinical examination. Rats with detectable deviations from the standard health indicators were excluded from the experimental groups.

Randomization of animals into groups (n = 6 in the group) was carried out by random selection using body weight as a leading parameter, so that the individual weight values did not deviate from the average by more than 10%. All records of behavioral reactions were carried out at the same time – in the evening, from 6 to 8 p.m. Spectra were recorded in the morning of the following day.

### 2.4 Experimental procedures

Animals of the exposed group were administered intragastrically with experimentally pure grade zinc succinate (PromSnab, Ltd., Russia) solution in deionized water in a single dose of 100 mg/kg. All the experimental evaluations were carried out 1 month after treatment.

### 2.5 Behavioral reactions

The study of motor activity in rats was performed on validated Laboras® device (Metris, the Netherlands), which is an automated, non-invasive system [13] for recognition and analysis of such behavioral responses as motion, immobility, vertical stand, grooming, water and food intake, locomotion as well as the parameters of motor activity (i.e. the distance travelled, speed of motion, etc.).

### 2.6 The study of the fluorescence parameters of the motor cortex

The animals were narcotized by intraperitoneal injection of Zoletil 100 (Vibrac, France) in a standard dose of 25 mg/kg. Operational access to the motor cortex of the cerebrum was carried out by making a window in the skull bone tissue and pre-fixing the head of the animal in the stereotaxic apparatus. A skin-aponeurosis flap with a portion of the skull bone was removed directly above the investigated part of the brain motor cortex. During the measurement, the studied zone of the brain was rinsed with a warm isotonic solution of NaCl.

For *in vivo* measurements, a multifunctional laser for non-invasive diagnostic system LAKK-M (SPE “LAZMA”, Ltd, Russia) with a measuring channel for fluorescence spectroscopy (FS) was used. The system provides multi-wavelength excitation of fluorescence at different wavelengths, records the fluorescence emission and processes the received signal. The fibre optical probe consisted of two multimode fibres for the fluorescence excitation and one fibre for the collection of the fluorescence emission. In the FS channel, the diameters of all the probing and receiving fibres were 400 μm. For safety reasons, as well as to keep photobleaching of the tissue at an acceptable level, probe radiation power of the 365-nm excitation wavelength at the output of the fibre probe did not exceed 1.5 mW. The output power for the 450-nm excitation wavelength did not exceed 3.5 mW. The source-detector spacing for the FS channel was 1 mm. The above-mentioned fluorescence excitation powers are provided at the tip of the fibre probe, which induces an excitation light flux in the tissue of no more than 0.16 W m^−2^ for 365 nm and 0.37 W m^−2^ for 450 nm. The numerical aperture of the fibres was 0.22. The spectrometer was equipped with a diffraction grating polychromator and a CCD-detector (TCD1304AP, Toshiba, Japan) [14]. During the recording, the rats were placed on a heating blanket so the temperature was maintained near 37 °C.

Signals from the measuring device were recorded on the surface of the rat motor cortex [15] with an interval of 5–10 s. For each hemisphere, 10 spectra were recorded. Each measurement did not take more than 2 s, with the brain tissues being exposed either to the UV or blue radiation. During such a short exposure interval, the photobleaching was considered to be negligible and accepted as having the same effect in all groups of the animals under study [16].

Excitation at 365 nm and 450 nm was used to induce fluorescence signals, which correspond to the NADH and FAD absorption maxima, respectively. Fluorescence changes of NADH and FAD are closely related to the blood flow [10, 17, 18] and cell metabolic state in most organs, as well as to the processes of tissue respiration [19, 20]. Selection of wavelengths was based on the fact that when excited in the biological tissues with a UV light (e.g., 365 nm), the endogenous NADH fluorescence is detected in the range of 490–510 nm [21]. When excited with blue light (e.g., at 430–450 nm), fluorescent flavins were recorded as emitting in the range of between 510 to 520 nm [22].

### 2.7 Morphological studies of organs and tissues

Following the analysis of the spectra of the motor cortex fluorescence, the animals were euthanized by inhalation of CO_2_ in the gas chamber, and subsequently brain, heart, lungs, liver and kidney samples were collected to perform morphological assessment of the intragastric zinc succinate administration effects on the organism. Organ samples were fixed in Carnoy's fluid and embedded in paraffin. 5 μm-thick sections were stained with 1% cresyl violet aqueous solution with acetate buffer (56 °C) for 20 min (for brain sections), and the other organs' specimen were stained with hematoxylin and eosin by standard methods [23].

### 2.8 Methods of statistical analysis

Statistical processing of the collected data was performed using the "Origin" software (OriginLab Corporation, USA). Median, interquartile scale (Me, 25L; 75U) and U Mann-Whitney tests were applied. The probability level of at least 95% was considered statistically significant (p ≤ 0.05).

## 3 Experimental results and discussion

### 3.1 Behavioral activity analysis

The results of the analysis of the rats’ behavioral activity 1 month after a single 100 mg/kg zinc succinate administration demonstrate a 3 to 5-fold decrease in the duration and speed of movement, distance traveled, as well as in the total number of registered behavioral acts compared to the control group (Table 1).

**Table 1:** The behavioral activity of rats registered over 20 min using a Laboras® system, Me (25L; 75U), n = 6 in a group.

Groups	Laboras® measured parameters			
	Duration of motor acts, s	Average speed, mm/s	Distance traveled, m	The total number of behavioral acts
1. Control animals group	6,5 (4,6; 9,4)	0,5 (0,5; 0,7)	0,6 (0,5; 0,8)	136,0 (109,3; 193,3)
2. Animal's obtained zinc succinate group	2,1∗ (0,7; 2,9)	0,1∗ (0,1; 0,2)	0,1∗ (0,1; 0,2)	40,5∗ (38,8; 44,1)

∗ *p ≤ 0,05, compared to controls*.

A decrease in motor activity was noted in all animals from the experimental group in comparison to the controls, which clearly indicates the inhibitory effect of intragastric administration of zinc succinate at the chosen concentration on the behavioral activity in rats.

### 3.2 Analysis of the fluorescence spectra in the brain cortex

**Figure 2:**
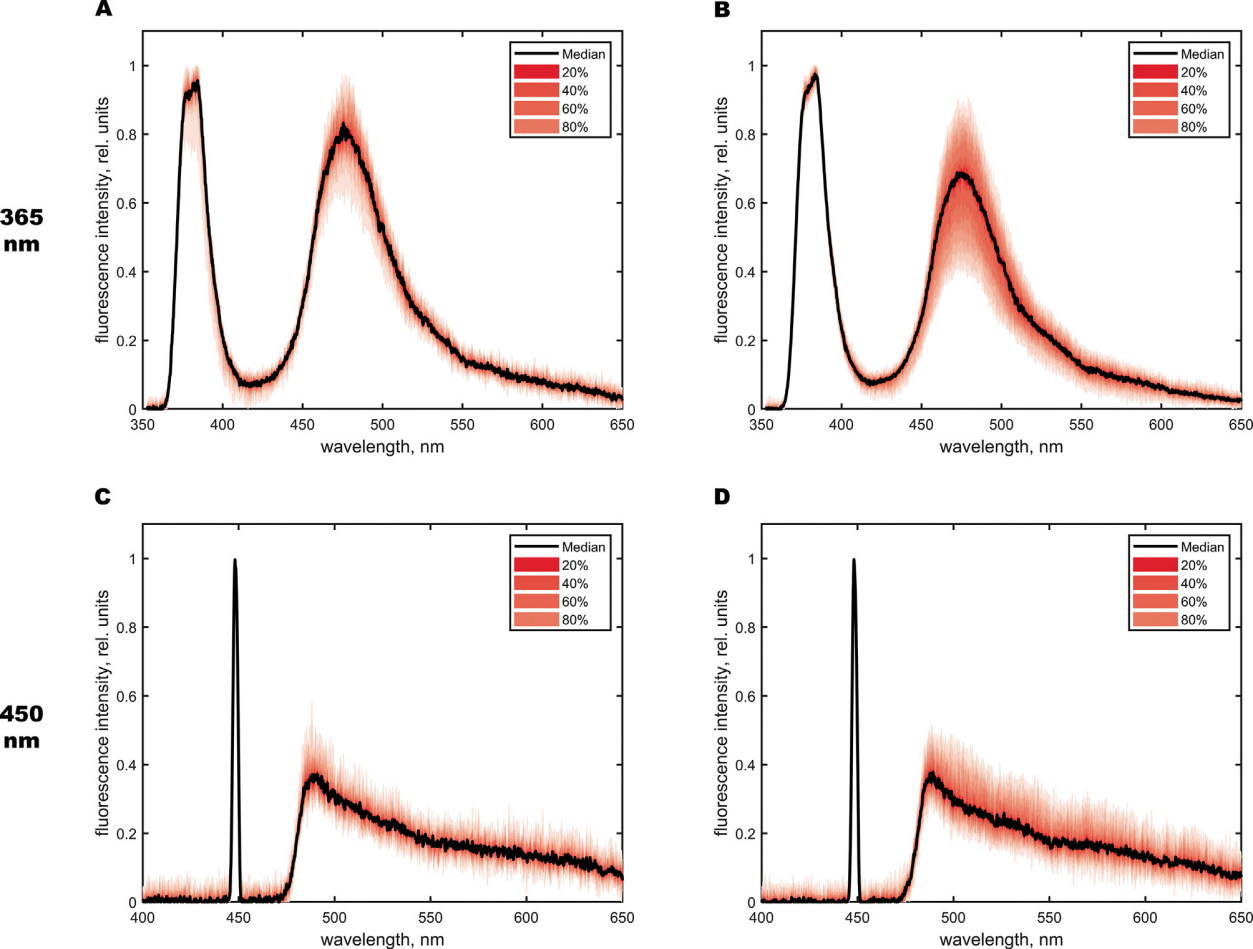
Fluorescence intensity for the control (A, C) group and the group that received zinc succinate (B, D) in the motor area of the cerebral cortex at 365 and 450 nm.

To analyze the functional activity of the motor cortex, which participates in the regulation of motor behavior in rats, spectra were recorded from its sections and for each hemisphere averaged for each group of animals. When constructing the distribution of the spectra, the median of the sample (Me) and its quartile percentage distribution were calculated and the maximal fluorescence intensity (I_f_) was chosen as the readout parameter. Figure 2 shows the obtained spectra indicating the fluorescence intensity. Spectral normalization in accordance with back-reflected radiation was achieved by dividing the spectrum of the intensity by the backscattered radiation (I_laser_).

**Figure 3:**
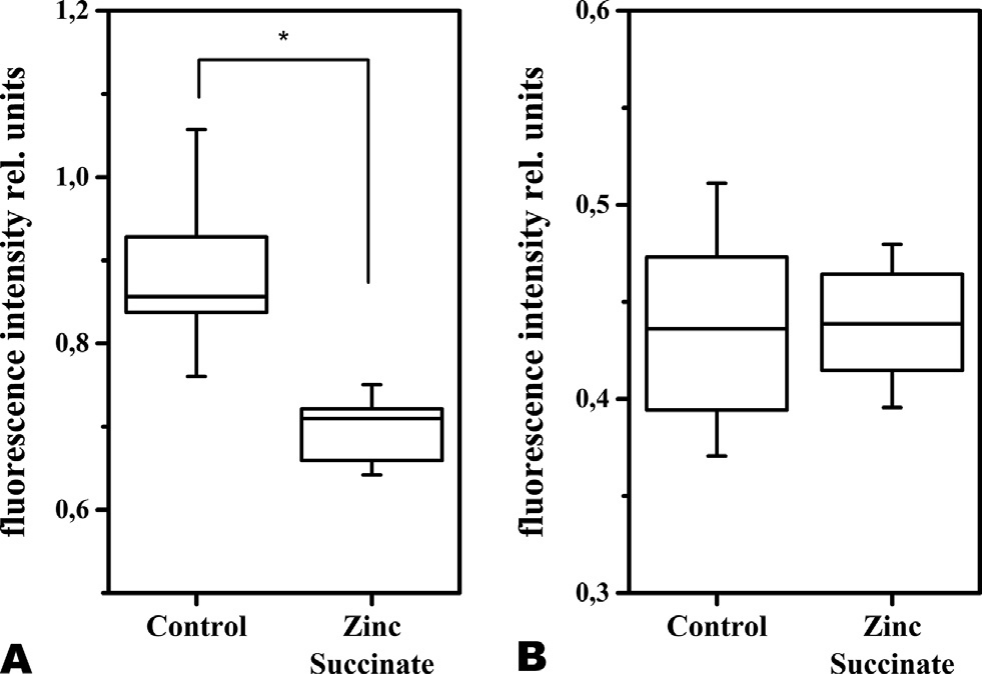
Diagrams of the maximum fluorescence intensity distribution for the wavelengths of 365 nm (A) and 450 nm (B) in the motor area of the cerebral cortex.

Statistically significant differences relative to the control group were confirmed using the Mann-Whitney test. The obtained corresponding fluorescence distribution diagrams are shown in Figure 3.

The presented results show that after intragastric zinc succinate administration, the fluorescence intensity at 365 nm decreases remarkably, whereas at a wavelength of 450 nm there was no significant change in registered fluorescence intensity, clearly indicating a reduction in coenzyme NADH without the decline of FAD levels. According to results of Genina et al. [24], in the studies of optical properties of brain tissues at the different stages of glioma development in rats, the diagnostics depth of the healthy cortex tissues in rats at the wavelengths of 365–420 nm reaches approximately 0.4–0.5 mm. For the used configuration of the fibre optical probe for such a diagnostic depth, the diagnostic volume can be assessed by Monte Carlo modelling at the level of 0.3–0.4 mm^3^ [16]. Since the signal depth was not more than 0.5 mm, 1–3 layers of the motor cortex were observed, in which small, medium and large pyramidal neurons, as well as glial cells and nerve fibers, are located [25].

### 3.3 Morphological analysis of the animals’ organs and tissues

After studying the behavioral reactions and analyzing the fluorescence spectra of the cerebral cortex, euthanasia and organ autopsy were performed in animals. The structural changes were investigated in the brain, heart, lungs, liver and kidneys. In the case of paired organs, both samples were examined. The corresponding organs of intact animals served as control.

Morphological analysis of the internal organs demonstrated pronounced structural changes in rats treated with zinc succinate in comparison to the intact animals.

**Figure 4:**
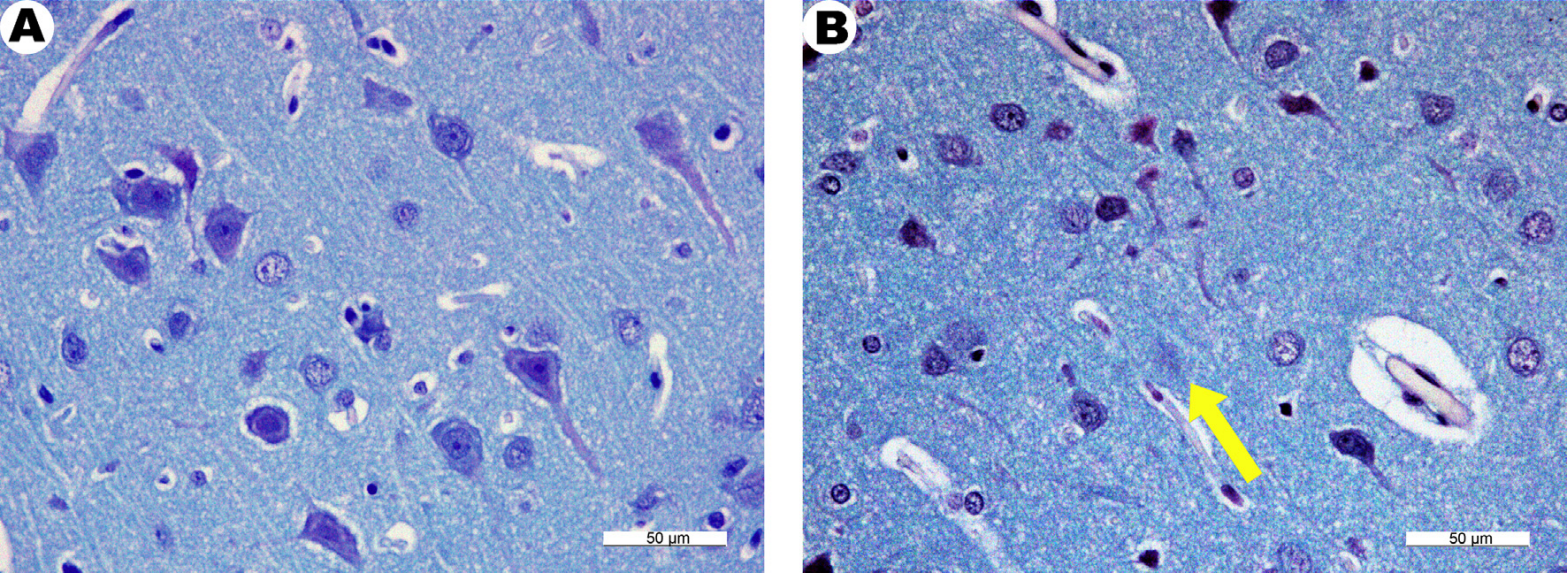
Fragments of the motor cortex of the cerebral hemispheres of control intact rats (A) and rats 1 month after receiving zinc succinate (B) stained with cresyl violet. (A), most neurons are pale stained with some signs of mild pericellular edema. (B), significantly pronounced pathological changes are detectable. The contours of the cells are indistinct (arrow). Most of the neurons appear to be in the necrobiosis state.

Thus, when the motor cortex was studied in intact animals, the neurons were predominantly of pyramidal form, their bodies in most cases look rounded, the cytoplasm was faintly stained and had the appearance of a narrow rim containing basophilic granules with poorly outlined contours (Figure 4A).

The analysis of the brain structures in animals treated with zinc succinate showed that the soft meninx could be traced in the form of small fragments, and the vessels were mostly full-blooded. In some areas, the expansion of the perivascular space was visualized. The overall brain histo-architecture was normal. Most of the neurons were stained pale, rounded, with a swelling noted in some of them, and a small number of neurons presented in the form of shadow cells. Small glial cells with hyperchromic, intensely stained nuclei were observed. No signs of neurophagy were observed (Figure 4B).

**Figure 5:**
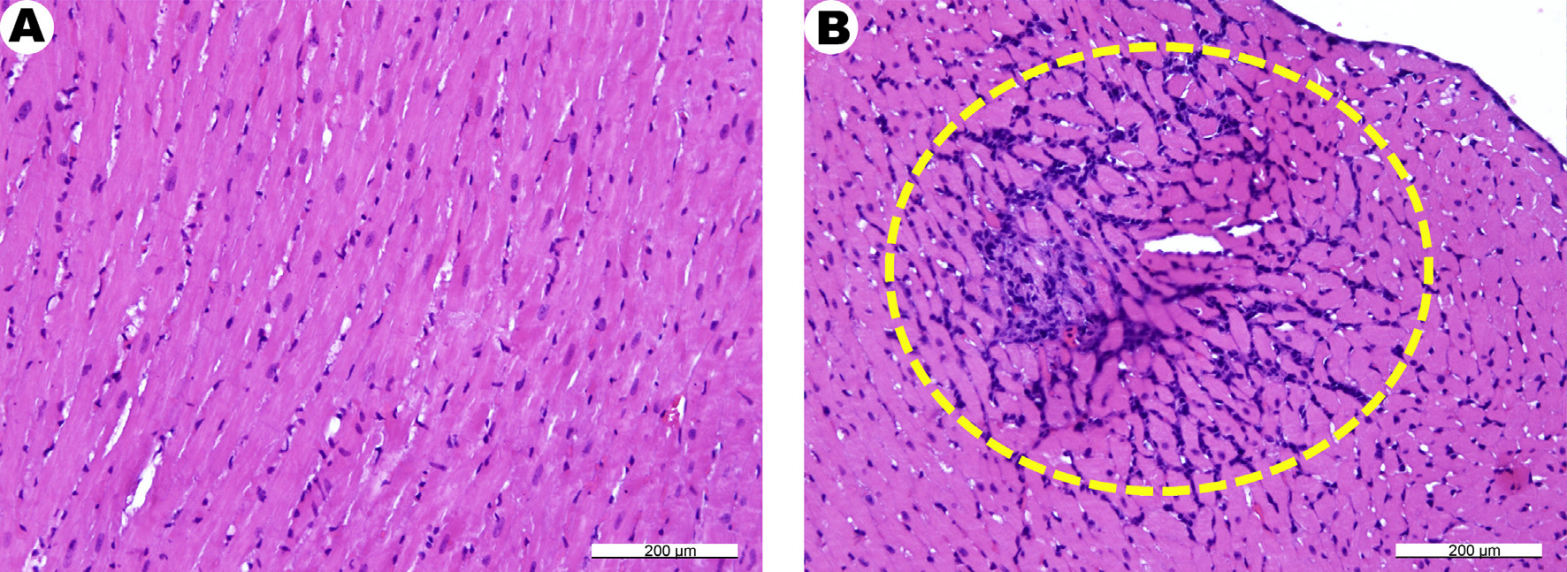
Rat myocardium fragments of intact control, rats (A) and rats 1 month after receiving zinc succinate (B). (A), normal microstructure of myocardium is seen. (B), the focus of muscle fibers necrosis (in oval) with the formation of lymphatic-macrophagal granuloma and the beginning of the sclerosis formation. H&E stain.

Morphological study of cardiac tissue in animals from the control group revealed uniform staining of cardiomyocytes. Their nuclei were not enlarged and had clear contours and most of them appeared of the same size and normochromic. The transverse striation of muscle fibers was observed in some fields of view (Figure 5A).

Cardiac tissue in animals of the experimental group treated by zinc succinate was characterized by an increase of dystrophic and altered cells in the myocardium, fragmentation of cardiomyocytes, omnidirectional myocardial fibers, single micro-focal changes and interstitial edema. The manifestations of protein dystrophy in individual cardiomyocytes, likely associated with hypoxia caused by the associated pathological conditions of the respiratory system (bronchitis, interstitial pneumonia and atelectasis), as well as uneven blood supply with single areas of ischemia were noted. The foci of sclerosis were often identified (Figure 5B).

**Figure 6:**
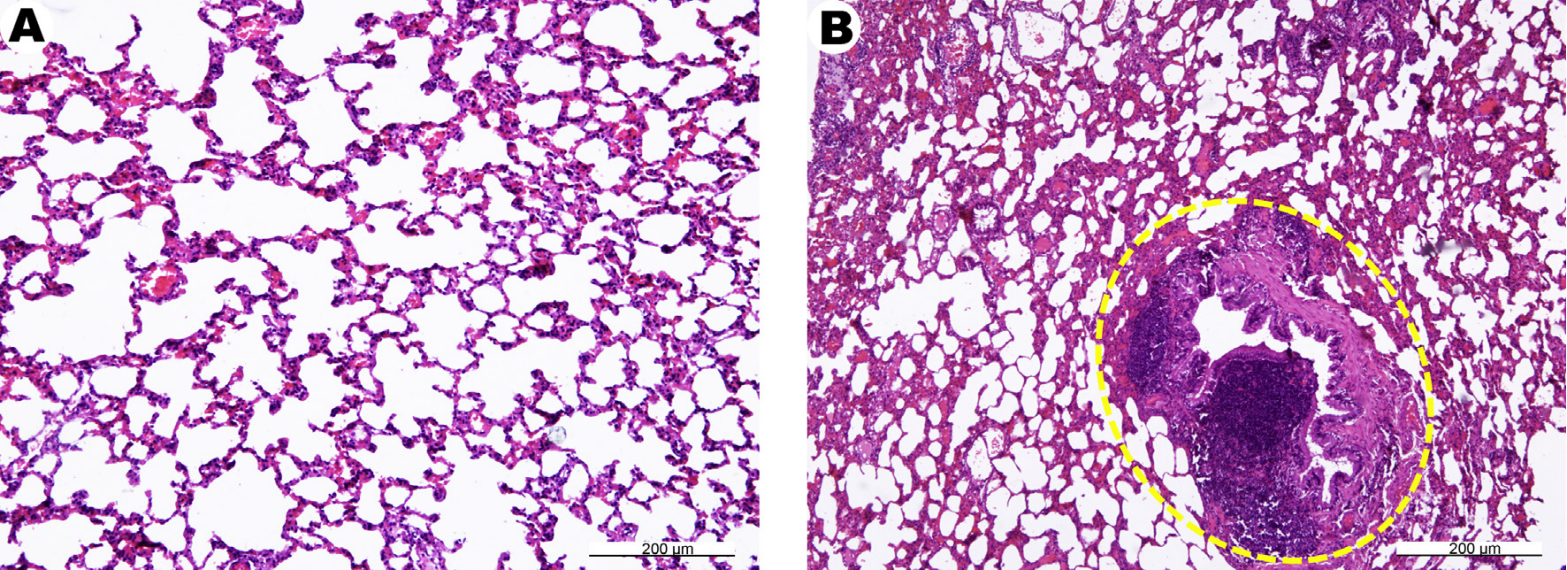
Microphotographs of the lungs of rats – intact control (A) and 1 month after receiving zinc succinate (B). (A), normal lung tissue microstructure is seen. (B), atelectasis of the lung tissue, small caliber bronchus with peribronchial lymphocytic infiltration (in oval). H&E stain.

In lung tissue specimens of the intact group, inter-alveolar septum and well-formed pulmonary parenchyma of normal thickness in the respiratory compartment was observed. A moderate expansion of the lumen of the alveoli and alveolar passages has been noted. The walls of the bronchi and bronchioles in almost all samples had a normal structure and were lined with a respiratory epithelium without any signs of inflammation, peribronchial edema or other pathological changes (Figure 6A).

In contrast, in the experimental group, the morphological signs of chronic bronchitis, small focal interstitial pneumonia, and emphysematous extended areas in the respiratory regions were registered in 2 out of 6 animals. Marked peribronchial lymphocytic proliferation, and an increased number of atelectatic and distelectatic sites was registered (Figure 6B).

**Figure 7:**
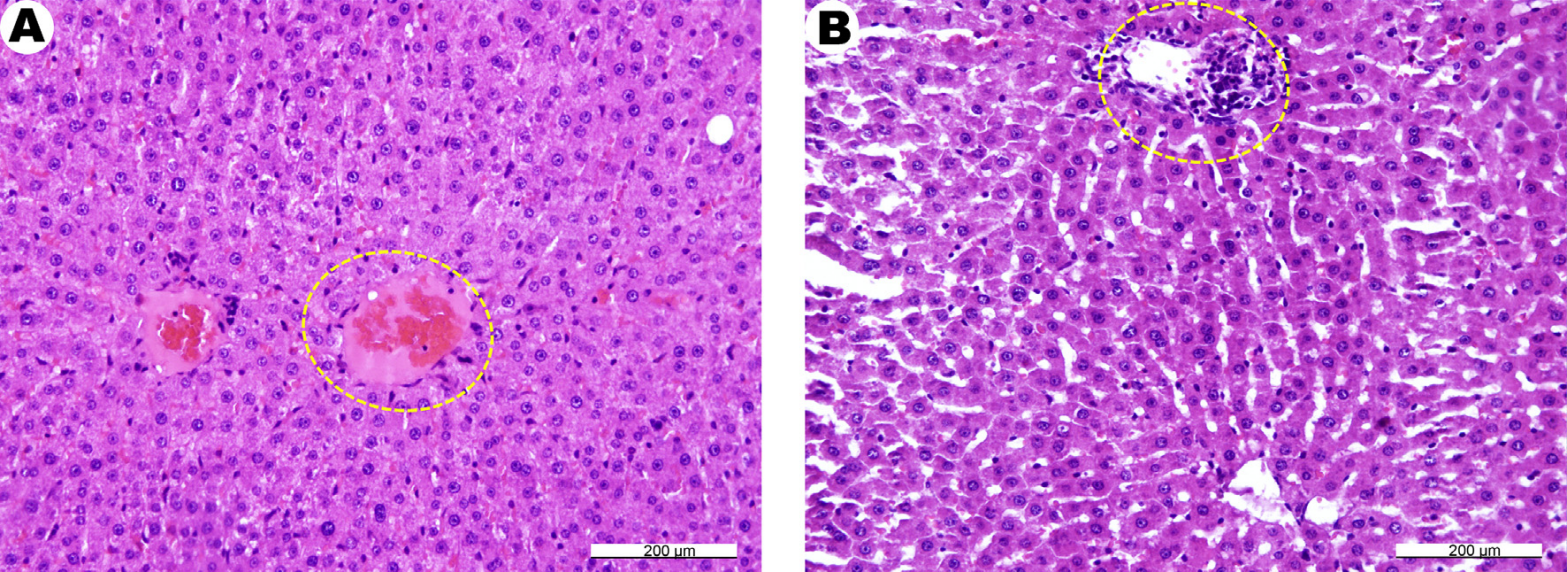
Fragments of the rat liver – intact control (A) and 1 month after receiving zinc succinate (B). (A), normal liver microstructure. There is a plethora of central veins and sinusoids (in oval). (B), focal necrosis of liver cells with lymphohistiocytic infiltration (in oval). H&E stain.

A study of the liver histological structure of intact animals showed the abundance of portal and central veins and normal lobular appearance. Hepatocytes of all the lobules had a normal structure without signs of protein granular dystrophy (Figure 7A). In the animals treated with zinc succinate, a number of sites with focal necrosis of liver cells and periportal lymphohistiocytic infiltrates was observed. The presence of dystrophic processes was also noted (Figure 7B).

**Figure 8:**
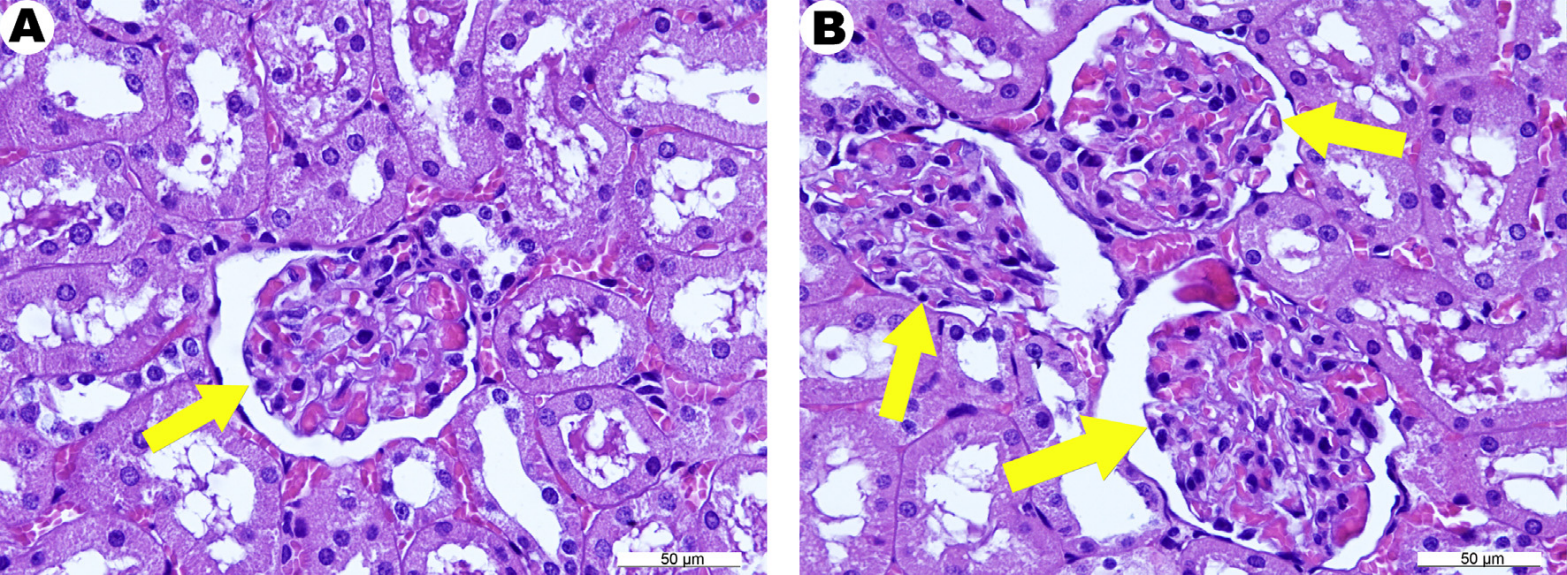
Fragments of the rat kidney – intact control (A) and 1 month after receiving zinc succinate (B). (A), fragment of the normal kidney tissue. One renal glomerulus is seen in the field of view (arrow). (B) the overall morphological picture of the kidney is similar to that shown in Panel A. Three renal glomeruli are seen in the field of view (arrows). Moderate granular dystrophy of the epithelium of the renal tubules. Capillaries of the renal glomeruli appear full-blooded. H&E stain.

Histological changes in the structure of the kidneys for both groups were not clearly detectable (Figures 8A, B). The glomeruli were predominantly of the same size, mostly full-blooded and beared no signs of pathological changes. The capsule of Shumlyansky-Bowman was not affected. The epithelium of the proximal and distal tubules was intact, with no pronounced dystrophic changes.

Thus, the morphological analysis of organs and tissues in rats treated with zinc succinate at a single intragastric dose of 100 mg/kg compared with animals of the control group clearly demonstrated toxic and dystrophic changes in the structures of the brain, heart, lungs and liver 1 month after treatment. No profound pathological changes in the kidneys were detected.

The results of our studies corroborate with the concept that a decrease in the fluorescence intensity of coenzyme NADH wavelength may indicate the suppression of metabolic processes in cells and tissues, as well as the cell death initiation [26]. Further development of this process can lead to the death of neurons. Of note, with short-term ischemia of cells, the fluorescence intensity of NADH increases, but in the conditions of a chronic experiment, the opposite effect is observed [27, 28].

At the same time, the accumulation of zinc succinate contributes to the damage of neurons in areas of the cerebral cortex in rats. Excess amounts of zinc concentrate in the mitochondria leads to their dysfunction, and as a result to the impair in tissue respiration processes [29]. Toxic doses of zinc succinate have been reported to cause mitochondrial depolarization and dysfunction [6].

Our study demonstrated a decrease in motor activity in rats of the experimental group, as well as a decrease in the fluorescence intensity of coenzyme NADH, while the fluorescence intensity of coenzyme FAD remained at the same level. The observed inhibition of tissue metabolism in the test areas, in accordance with the previously published reports [7, 10, 30], may lead to irreversible pathological changes. The data presented here may indicate the initiation of mitochondrial dysfunction and disruption of the processes of oxygenation of neurons ultimately leading to their death, which corresponds to the results of the evaluation of the behavior of rats. The data of the morphological analysis indicate a toxic effect of zinc succinate on the structure of the brain, heart, lungs and liver of the experimental animals, without any detectable pathological changes in the kidneys.

## 4 Conclusions

The presented original complex morphofunctional approach to assess the toxic effects of the metal salts on the structure of the organs and tissues in general and on the brain in particular, convincingly allows the detection of both functional and structural toxic effects of zinc compounds and permits to evaluate the redox process in cerebral cortex structures of the exposed rats. Of note, this approach enables to register toxicity manifestations after a single dose oral administration of zinc succinate at the dose up to ten times lower than the acute LD_50_ for the common zinc salts established through the International Programme on Chemical Safety conducted by the World Health Organization [31]. Application of both physiological and advanced microscopy methods of diagnostics may be used to develop new strategies for assessing breakdowns of metabolic processes in the structures of the brain cortex in laboratory animals in general toxicology studies, as well as in the studies of pathological conditions affecting the central nervous system, since the manifestations of the toxic effect of zinc succinate on brain structures and behavioral characteristics of animals are similar to the clinical signs of some neurodegenerative diseases, e.g. in the Alzheimer's disease model [3].

## Declarations

### Author contribution statement

Y. Volkov, A. Alekseev and S. Kuznetsov: Conceived and designed the experiments; Analyzed and interpreted the data.

G. Piavchenko: Performed the experiments; Analyzed and interpreted the data; Wrote the paper.

O. Stelmashchuk, E. Seryogina, A. Dunaev, E. Zherebstov and E. Kuznetsova: Performed the experiments; Contributed reagents, materials, analysis tools or data.

### Funding statement

The study was funded by Russian Foundation for Basic Research (Russian Federation), Research Project № 18-02-00669. E. Zherebtsov acknowledges funding from the grant of the Academy of Finland (grant No. 318281), A. Dunaev also acknowledges funding from the Academy of Finland (grant No. 326204).

### Competing interest statement

The authors declare no conflict of interest.

### Additional information

No additional information is available for this paper.
